# Propolis Augments Apoptosis Induced by Butyrate via Targeting Cell Survival Pathways

**DOI:** 10.1371/journal.pone.0073151

**Published:** 2013-09-04

**Authors:** Eric Drago, Michael Bordonaro, Seon Lee, Wafa Atamna, Darina L. Lazarova

**Affiliations:** Department of Basic Sciences, The Commonwealth Medical College, Scranton, Pennsylvania, United States of America; Van Andel Institute, United States of America

## Abstract

Diet is one of the major lifestyle factors affecting incidence of colorectal cancer (CC), and despite accumulating evidence that numerous diet-derived compounds modulate CC incidence, definitive dietary recommendations are not available. We propose a strategy that could facilitate the design of dietary supplements with CC-preventive properties. Thus, nutrient combinations that are a source of apoptosis-inducers and inhibitors of compensatory cell proliferation pathways (e.g., AKT signaling) may produce high levels of programmed death in CC cells. Here we report the combined effect of butyrate, an apoptosis inducer that is produced through fermentation of fiber in the colon, and propolis, a honeybee product, on CC cells. We established that propolis increases the apoptosis of CC cells exposed to butyrate through suppression of cell survival pathways such as the AKT signaling. The programmed death of CC cells by combined exposure to butyrate and propolis is further augmented by inhibition of the JNK signaling pathway. Analyses on the contribution of the downstream targets of JNK signaling, c-JUN and JAK/STAT, to the apoptosis of butyrate/propolis-treated CC cells ascertained that JAK/STAT signaling has an anti-apoptotic role; whereas, the role of cJUN might be dependent upon regulatory cell factors. Thus, our studies ascertained that propolis augments apoptosis of butyrate-sensitive CC cells and re-sensitizes butyrate-resistant CC cells to apoptosis by suppressing AKT signaling and downregulating the JAK/STAT pathway. Future *in vivo* studies should evaluate the CC-preventive potential of a dietary supplement that produces high levels of colonic butyrate, propolis, and diet-derived JAK/STAT inhibitors.

## Introduction

Butyrate, a fermentation product of fiber in the colon, is a histone deacetylase inhibitor (HDACi) that induces apoptosis in colon cancer (CC) cells with mutations in the WNT/beta-catenin pathway [1], [2]. We have previously reported that one mechanism by which butyrate induces high levels of apoptosis of such CC cells is through hyperactivation of WNT/beta-catenin signaling, and this activity is mimicked by structurally unrelated HDACis [1], [2]. The apoptotic levels in CC cell populations exposed to HDACis are limited by the induction of cell survival pathways. HDACi-treated apoptotic CC cell populations exhibit augmented AKT cell survival signaling, EGFR signaling, and express immediately-early genes *FOS* and *JUN* that can promote cell proliferation [3]–[9]. The induction of cell survival mechanisms in apoptotic CC cell populations is reminiscent of compensatory proliferation, a phenomenon first observed in *Drosophila* tissues where massive cell death is followed by proliferation that compensates for lost cells. The proliferation is triggered by apoptotic *Drosophila* cells that secrete homologs of TGFbeta and WNT ligands, mitogens that support the recovery of the remaining living cells [10]–[15]. The phenomenon is not limited to *Drosophila*, as different types of human tissue including that of the gut regenerate through apoptosis-induced compensatory proliferation [13]. Therefore, it is not surprising that CC cell populations undergoing HDACi-triggered apoptosis induce the cell survival AKT pathway, and this induction is likely triggered by increased expression of TGFbeta and several WNT ligands [3], [16].

Consistent with the finding that HDACi-induced apoptosis of CC cell populations is accompanied by increasing AKT signaling in the surviving cells, we have observed that the combination of synthetic HDACis and inhibitors of AKT kinase (AKTis) augments apoptosis [3]. The strategy of combining a HDACi and an AKTi can be applied not only in CC therapeutics, but also in CC prevention since some dietary compounds have the ability to inhibit histone deacetylases and AKT [3]. Butyrate is the most potent diet-derived HDACi, and its concentration in the colon reaches 5 to 10 mM after intake of dietary fiber [17]. Several diet-derived compounds have been reported to inhibit the activation (phosphorylation) of AKT kinase, and we have established that diallyl trisulfide, a garlic-derived compound, and caffeic acid phenethyl ester (CAPE), a compound contained in some honeybee propolis supplements, suppress the induction of phosphorylated (p) AKT levels in butyrate-treated CC cells [3]. Therefore, the simultaneous intake of dietary fiber, resulting in colonic butyrate, and diet-derived inhibitors of pAKT may induce high levels of apoptosis in neoplastic colonic cells. To take the first step in testing this hypothesis, we ascertained the ability of CAPE-containing propolis, an available dietary supplement, to augment the apoptotic effect of butyrate in CC cells. We report that: (a) CAPE, and a propolis preparation that contains equivalent or lower levels of CAPE (propolis with CYCLOPOWER ™, Manuka Health New Zealand Ltd), exert differential effects on apoptosis in butyrate-treated CC cells, and (b) the increased apoptosis of CC cells exposed to butyrate and propolis is due to the suppression of AKT signaling and downregulation of the JAK/STAT pathway. Based upon these findings, we posit that a dietary supplement that produces high levels of butyrate and propolis in the colon has CC-preventive properties.

## Results

### The Apoptotic Effect of Butyrate on CC Cells is Augmented to Different Extent by CAPE and Propolis

We have reported that CAPE suppresses the activation of the AKT survival pathway in CC cells exposed to butyrate [3]. In that previous study, among the analyzed CC cell lines were HCT-116 cells, which are butyrate-sensitive and undergo approximately 50% apoptosis after 24 hours of exposure to 5 mM butyrate, and butyrate-resistant HCT-R cells, which are routinely grown in cell culture medium with 5 mM butyrate. HCT-R cells were derived from HCT-116 cells by a prolonged exposure to increasing levels of butyrate [2], and can be re-sensitized to the apoptotic effect of butyrate through co-treatment with 4 µg/ml CAPE [3].

**Figure 1:**
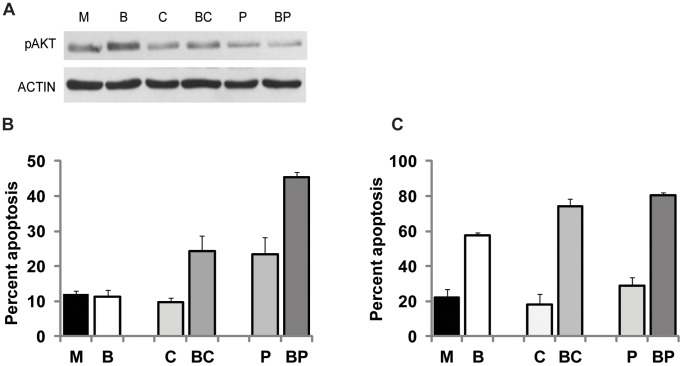
CAPE-containing propolis augments the apoptotic effect of butyrate on CC cells. (A). Representative western blot analysis of HCT-R cells exposed to mock (M), 5 mM butyrate (B), 1.2 µg/ml CAPE, butyrate and CAPE (BC), 100 µg/ml propolis (P), or butyrate and propolis (BP) for 19 h. (B, C) HCT-R (B) and HCT-116 (C) cells were exposed to mock (M), 5 mM butyrate (B), 1.2 µg/ml CAPE (C), butyrate and CAPE (BC), 100 µg/ml propolis (P), or butyrate and propolis (BP) for 50 h. Propolis preparation contains 0.3 µg CAPE per 100 µg powder. A minimum three independent experiments were carried out with triplicate samples each, values are mean ± SD.

We utilized HCT-116 and HCT-R cells to compare the apoptotic effect of CAPE to that of propolis. First, we evaluated the concentration of CAPE that suppresses phosphorylated (p) AKT levels to the same extent as 100 µg/ml propolis. Western blot analyses revealed that 1.2 µg/ml CAPE and 100 µg/ml propolis decrease pAKT levels in HCT-R CC cells to the same extent (Fig. 1A). Since 100 µg of propolis contain only 0.3 µg of CAPE, it is likely that in addition to CAPE, other propolis-specific compounds inhibit the pAKT levels in butyrate-treated CC cells.

The higher biological activity of propolis compared to that of CAPE was ascertained by apoptotic assays (Figs. 1B and 1C). HCT-116 cells (Fig. 1C) exposed to mock treatment, CAPE, or propolis did not undergo significant levels of apoptosis (22.3±4.4, 18.3±5.8, and 28.7±4.9, respectively). However, adding CAPE or propolis to butyrate augmented the apoptotic levels of HCT-116 cells compared to the apoptosis induced by butyrate alone (74.0±4.3 and 80.5±1.4 compared to 57.5±1.5, P<0.05). HCT-R cells (Fig. 1B) exposed to mock treatment or CAPE did not undergo significant levels of apoptosis (12.1±1.0 and 9.7±1.2); however, exposure to propolis increased apoptosis significantly (23.4±4.9%, P<0.05). Addition of CAPE or propolis to butyrate re-sensitized HCT-R cells to the apoptotic effect of butyrate: butyrate-treated cells exhibited 11.2±2.1% apoptosis; whereas addition of CAPE or propolis resulted in 24.3±4.3% and 45.5±1.4%, respectively, P<0.05. The combination of butyrate and propolis exhibited more powerful apoptotic effect than the combination of butyrate and CAPE in HCT-R cells, and treatment with propolis alone induced low levels of apoptosis in HCT-R, but not in HCT-116 cells (Figs. 1A and 1B). Since 1.2 µg/ml CAPE exhibited less potent effect on HCT-R cells than propolis at 100 µg/ml (resulting in 0.3 µg/ml CAPE), we posited that in addition to CAPE, propolis contains compounds that target molecule(s) other than AKT.

### The Exposure of CC Cells to Butyrate and Propolis Induces the JNK Pathway

**Figure 2:**
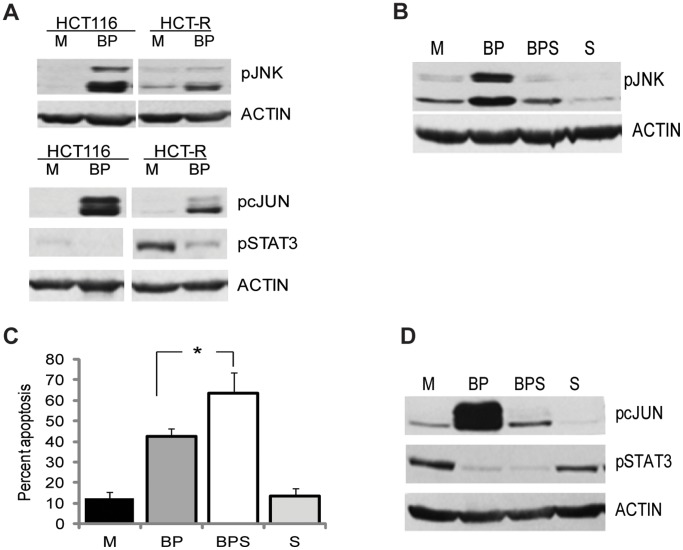
Role of JNK signaling in the apoptotic response of HCT-R cells to butyrate and propolis. (A). A representative western blot analysis of HCT-116 and HCT-R cells exposed to mock (M), or 5 mM butyrate and 100 µg/ml propolis (BP) for 19 h. (B) A representative immunoblot analysis of HCT-R cells exposed to mock (M), or 5 mM butyrate and 100 µg/ml propolis (BP), 20 µM SP600125 (S) or SP600125 and butyrate/propolis (BPS) for 19 h. (C) Suppression of JNK signaling augments apoptosis of HCT-R cells exposed to butyrate and propolis. HCT-R cells were exposed for 50 h to mock (M), 20 µM SP600125 (S), the combination of 5 mM butyrate and 100 µg/ml propolis (BP), or SP600125, butyrate, and propolis (BPS). Apoptosis was measured by flow cytometry with PE Annexin V Apoptosis Detection Kit I (BD Biosciences, #559763). A minimum three independent experiments were carried out with triplicate samples each, values are mean ± SD. (D) A representative western blot analysis of HCT-R cells treated as in (B), and developed with antibodies to the phosphorylated forms of cJUN and STAT3.

HCT-116 and HCT-R cells express high levels of WNT5A [3], and this WNT ligand can activate not only AKT, but also JNK signaling [18], [19]. Unlike the anti-apoptotic role of AKT signaling [3], the activation of JNK pathway has been reported to contribute to butyrate-induced apoptosis of CC cells without mutations in WNT/beta-catenin signaling [20]. Congruently, suppression of JNK signaling by NOTCH signaling confers resistance to apoptosis in non-transformed breast epithelial cells [21]. Based upon these data, we ascertained whether propolis augments butyrate-induced apoptosis of HCT-R cells through activation of JNK signaling. Western blot analyses revealed that JNK signaling was induced in butyrate/propolis-treated cells compared to mock-treated cells (Fig. 2A). Consistent with this finding, c-JUN, a main target of JNK signaling, was phosphorylated in cells exposed to butyrate and propolis (Fig. 2A). In addition to activating c-JUN, JNK signaling initiates the release of cytokines that induce the JAK/STAT pathway [22], [23]. Therefore, we determined whether co-treatment of CC cells with butyrate and propolis affects the levels of phosphorylated (p) STAT3, a component of the JAK/STAT pathway. Western blot analyses revealed that HCT-R cells express higher levels of pSTAT3 than HCT-116 cells, and that exposure to butyrate/propolis downregulates, but does not eliminate the levels of pSTAT3 in the drug-resistant CC cells (Fig. 2A).

To determine whether the increase in JNK activity contributes to the apoptotic effect of butyrate and propolis in HCT-R CC cells, we utilized SP600125, a reversible ATP competitive inhibitor of the JNK enzymes [24]. First, we established that the inhibitor downregulates the activation of JNK in HCT-R cells exposed to butyrate and propolis (Fig. 2B), and then we evaluated how the suppression of pJNK levels affects apoptosis in butyrate/propolis-treated HCT-R cells. Contrary to studies with other cell types [20], [21], the pharmacological suppression of JNK activity in HCT-R CC cells augmented apoptosis induced by butyrate and propolis (Fig. 2C). Exposure to mock or SP600125 treatment resulted in 12.3±3.3% and 13.6±0.3% apoptosis, respectively. Co-treatment of the cells with butyrate and propolis produced 42.5±3.8% apoptosis, and addition of SP600125 increased apoptosis to 63.4±10.1%, P<0.05. Western blot analyses revealed that the suppression of pJNK by the inhibitor SP600125 downregulates the levels of the two downstream targets pcJUN and pSTAT3 (Fig. 2D).

### Role for Phosphorylated (p) cJUN in the Apoptosis of Butyrate/Propolis-treated CC Cells

Inhibition of pJNK levels suppresses the activity of its downstream targets, pcJUN and pSTAT3, and augments apoptosis of butyrate/propolis-treated CC cells (Figs. 2C and 2D); therefore, we investigated the role of pcJUN and pSTAT3 in the apoptotic event.

**Figure 3:**
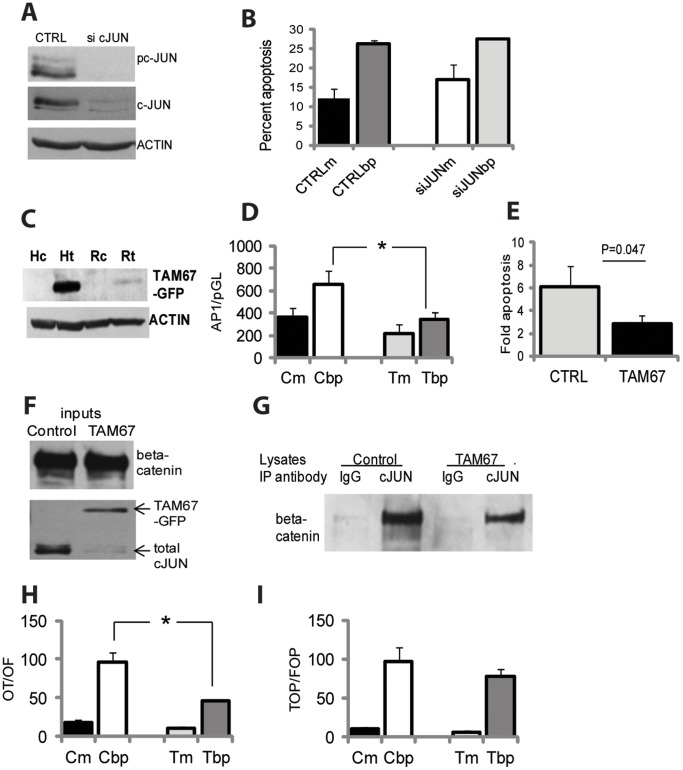
Role of cJUN in the apoptosis of butyrate/propolis-treated HCT-R cells. (A) Silencing of c*JUN* expression via nucleofection of c*JUN* siRNA suppresses total and phosphorylated (p) cJUN levels in HCT-R cells, as ascertained by western blot analyses. (B) A representative apoptotic analysis of HCT-R cells that were nucleofected with control or c*JUN* siRNA. Cells were exposed to mock or butyrate/propolis treatment for 24 h, and analyzed at 48 h post-nucleofection. Each treatment was in triplicate, values are mean ± SD. (C) Detection of TAM67-GFP in HCT-116 (H) or HCT-R (R) cells transfected stably with control (c) or TAM67 (t) expression vector. (D) AP1 luciferase transcription assays of control (C) and TAM67 (T) cells exposed for 19 h to mock (m) or 5 mM butyrate and 100 µg/ml propolis (bp) treatment. (E) Apoptotic assays of control and TAM67-expressing HCT-116 cells exposed for 24 h to mock or 5 mM butyrate and 100 µg/ml propolis were carried out as described in Methods. Three independent experiments were carried out with triplicate samples each, values are mean ± SD. (F) Expression of TAM67-GFP, endogenous cJUN, and beta-catenin in nuclear lysates of HCT-116 cells transfected with GFP (control) or TAM67-GFP vector. Cells were exposed to butyrate/propolis as in Fig. 1A for 19 h. TAM67 and endogenous cJUN were visualized with an antibody to the DNA-binding domain of c-JUN (sc-44, Santa Cruz Biotechnology). (G) Immunoprecipitations with nuclear lysates described in (F) with a control (IgG) antibody or a cJUN antibody that recognizes the amino-terminal domain of the endogenous cJUN protein (sc-45, Santa Cruz Biotechnology). Beta-catenin that co-immunoprecipitates with endogenous cJUN was labeled with an antibody from Santa Cruz Biotechnology (sc-53483). (H,I) Luciferase transcription assays of control (C) and TAM67 (T) cells exposed for 19 h to mock (m) or 5 mM butyrate and 100 µg/ml propolis (bp) were carried out as described in Methods. Three independent experiments were carried out with duplicate samples each, values are mean ± SD.

To determine how pcJUN affects apoptosis in butyrate/propolis-treated CC cells, we silenced *cJUN* expression with short interfering (si) RNAs. Western blot analyses ascertained effective downregulation of total and phosphorylated cJUN levels in HCT-R cells (Fig. 3A). Apoptotic assays with control and *cJUN* siRNA-transfected HCT-R cells established that the decrease in pcJUN protein levels does not affect apoptosis when the cells are exposed to butyrate/propolis treatment (Fig. 3B). Suppression of cJUN levels in HCT-116 cells similarly does not affect the levels of apoptosis induced by butyrate/propolis (data not shown).

In an alternative approach to elucidate the role of pcJUN, we transfected HCT-116 and HCT-R cells with a dominant negative (dn) form of cJUN (TAM67). TAM67 lacks the transactivation domain of cJUN (the N-terminal domain comprised of aa 3–122), but retains the DNA-binding and leucine zipper (dimerization) domains; therefore, the mutant protein diminishes the transcriptional activity that is dependent upon the N-terminal phosphorylation of cJUN. We reasoned that if the JNK inhibitor augments butyrate/propolis-induced apoptosis (Fig. 2C) by suppressing pcJUN levels, then TAM67 should mimic the effect of the JNK inhibitor. However, if the inhibition of JNK signaling augments apoptosis not through pcJUN activity, then apoptotic levels of TAM67-expressing cells may not differ from these of control transfected cells. Analyses of HCT-116 and HCT-R cells stably transfected with a TAM67-GFP vector established that HCT-116 cells express detectable levels of TAM67; whereas, HCT-R cells express the recombinant protein at relatively low levels (Fig. 3C). Therefore, we continued analyses on the effects of TAM67 in HCT-116 cells. To determine whether TAM67 is functional, and it suppresses AP1 transcriptional activity, we utilized a luciferase transcriptional assay with an (AP1)_4_ -luciferase reporter vector and a control pGL3Basic vector. These transcriptional reporter assays established that in absence of treatment (when the levels of pcJUN are relatively low, Fig. 2A), TAM67 does not affect significantly AP1 transcriptional activity (Fig. 3D). In the presence of butyrate/propolis (when the levels of pcJUN are increased, Fig. 2A), TAM67 expression suppresses AP1-dependent activity: control HCT116 cells exhibited an AP1/pGL3 ratio of 651.0±123.0, and TAM67 cells exhibited a ratio of 339.0±62.3, P = 0.023 (Fig. 3E). Unexpectedly, apoptotic assays established that the expression of TAM67 counteracts the apoptosis of butyrate/propolis-treated HCT-116 cells (Fig. 3D). The fold induction of apoptosis by butyrate/propolis treatment in control HCT-116 cells was 6.1±1.8; whereas, in TAM67-expressing cells the fold induction of apoptosis was 2.8±0.7, P = 0.048 (Fig. 3E).

Since apoptotic levels of CC cells exposed to butyrate depend upon the hyperinduction of WNT/beta-catenin activity [1]–[3], we hypothesized that the suppression of apoptosis by TAM67 may reflect its inhibitory effect on WNT/beta-catenin dependent transcription. TAM67 may downregulate WNT transcriptional activity (the activity mediated by beta-catenin/TCF4 complexes), since (a) cJUN binds to TCF4 and induces transcription of genes with proximal LEF/TCF and AP1 motifs [25], and (b) the leucine zipper domain of cJUN binds beta-catenin and transactivates genes with TCF-binding sites independently of AP1 motifs [26]. First, we ascertained whether the expression of TAM67 affects the binding of endogenous cJUN to beta-catenin and TCF4. Nuclear lysates of control GFP- and TAM67GFP-transfected cells (Fig. 3F) were immunoprecipitated with an antibody to the amino-terminal domain of cJUN. We were unable to detect binding between endogenous cJUN and TCF4; however, we observed that an antibody that recognizes endogenous cJUN, but not TAM67, co-immunoprecipitates beta-catenin (Fig. 3G). The amount of beta-catenin bound to endogenous cJUN in TAM67-transfected cells was consistently lower than this in control GFP-transfected cells. Next, we determined whether the decreased binding between endogenous cJUN and beta-catenin in TAM67-expressing cells affects the expression of WNT/catenin-dependent genes. We utilized two pairs of reporters that measure the transcriptional activity of WNT signaling-dependent complexes between beta-catenin and TCF4: TOPFlash/FOPFlash and LEF-OT/LEF-OF. In mock-treated cells, we found that the expression of TAM67 does not affect WNT/catenin transcriptional activity; whereas, in butyrate/propolis-treated cells, TAM67 significantly suppresses the induction of LEF-OT, a reporter with TCF-binding motifs. Thus, control cells expressed a LEF-OT/LEF-OF ratio of 96.3±11.8; whereas, TAM67 cells expressed a ratio of 45.8±0.5, P = 0.002 (Fig. 3H). Finally, TAM67 modulates in a statistically insignificant manner the induction of TOPFlash, a reporter with TCF and AP1 motifs: control cells expressed a TOPFlash/FOPFlash ratio of 96.4±18.5, and TAM67 cells exhibited a ratio of 77.1±10.0, P = 0.187 (Fig. 3I).

### Role for JAK/STAT Signaling in the Apoptosis of Butyrate/Propolis-treated CC Cells

**Figure 4:**
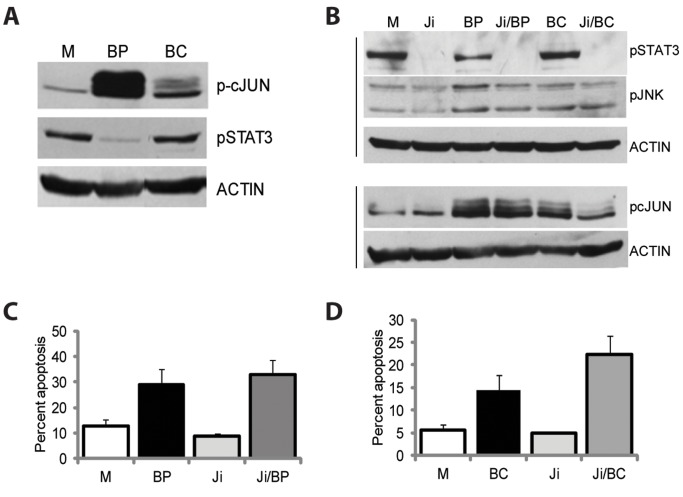
Role of JAK/STAT signaling in the apoptosis of butyrate/propolis-treated HCT-R cells. (A). A representative western blot analysis of HCT-R cells exposed to mock (M), 5 mM butyrate and 100 µg/ml propolis treatment (BP), 5 mM butyrate and 1.2 µg/ml CAPE (BC) for 19 h. (B). A representative immunoblot of HCT-R cells exposed for 19 h to mock (M), 1 µM JAK inhibitor pyridone 6 (Ji), 5 mM butyrate and 100 µg/ml propolis treatment (BP), the combination of JAK inhibitor, butyrate, and propolis (Ji/BP), 5 mM butyrate and 1.2 µg/ml CAPE (BC), and the combination of JAK inhibitor and butyrate/CAPE (Ji/BC). (C and D) Apoptotic analyses of HCT-R cells exposed for 35 hours to (M), 5 mM butyrate and 100 µg/ml propolis treatment (BP), 1 µM JAK inhibitor (Ji), 5 mM butyrate and 1.2 µg/ml CAPE (BC), JAK inhibitor and butyrate/propolis (Ji/BP), or JAK inhibitor and butyrate/CAPE (Ji/BC). A minimum of three independent experiments were carried out with triplicate samples each, values are mean ± SD.

Western blot analyses established that in absence of treatment, drug-resistant HCT-R cells express higher levels of pSTAT3 than drug-sensitive HCT-116 cells (Fig. 2A). Comparison between butyrate/CAPE- and butyrate/propolis-treated HCT-R cells revealed that whereas addition of propolis suppresses pSTAT3 levels, addition of CAPE does not (Fig. 4A). Therefore, we decided to compare the apoptotic effect of inhibited JAK/STAT signaling in CC cells exposed to butyrate/CAPE or butyrate/propolis. The rational was that if propolis augments butyrate-induced apoptosis more potently than CAPE by suppressing JAK/STAT signaling, then inhibiting JAK/STAT activity in butyrate/CAPE-exposed cells should result in apoptotic levels similar to these in butyrate/propolis-treated cells. The inhibition of JAK/STAT signaling was achieved with pyridone 6 (JAKi) [27]. At one micromolar concentration, this JAK/STAT inhibitor suppresses effectively the phosphorylated levels of STAT3 without affecting significantly the levels of pJNK (Fig. 4B). As expected, the addition of a JAK/STAT inhibitor to butyrate/propolis-treated cells did not result in a significant increase in the apoptotic levels: at 35 hours of treatment, butyrate/propolis-treated cells exhibited 29±5.9% of apoptosis; whereas, HCT-R cells exposed to butyrate/propolis/JAKi produced 32.7±5.7% apoptosis, P = 0.05 (Fig. 4C). The addition of a JAK inhibitor to butyrate/CAPE-treated HCT-R cells also did not result in a statistically significant increase in apoptosis: at 35 h of exposure, butyrate/CAPE-treated cells exhibited 14.5±3.2% apoptosis, whereas the cells exposed to butyrate/CAPE/JAKi exhibited 22.3±4.2% apoptosis, P = 0.06 (Fig. 4D).

Since propolis effectively downregulates pSTAT3 levels (Fig. 2A) and augments butyrate-induced apoptosis more effectively than CAPE, we tried to determine the individual propolis compounds that contribute to these effects. The analyzed by us Manuka Health CYCLOPOWER ™ propolis (New Zealand), as well as any other propolis preparations from New Zealand have been categorized as a European type propolis, i.e. derived largely from poplar, willow, and birch. A compositional comparison between New Zealand propolis and propolis preparations from other geographic regions including Europe, Brazil, and China have been carried out by Kumazawa *et al*., [28], and the major flavonoids and caffeic acid-based compounds of the New Zealand propolis has been reported by Markham *et al*., [29]. Thus, it is known that in addition to CAPE, the New Zealand propolis contains chrysin, galangin, and pinocembrin, all of which have variable effects on cellular systems *in vitro* [30]–[35]. Therefore, we ascertained whether these compounds contribute to the enhanced effect of propolis on butyrate-induced apoptosis of CC cells. We analyzed the individual compounds at levels equivalent to their concentrations in 100 µg propolis/ml cell culture medium. Consequently, the pro-apoptotic effect of chrysin, pinocembrin and galangin on butyrate-treated CC cells was evaluated at 3, 7, and 2 µM, respectively. None of the compounds re-sensitized HCT-R cells to the apoptotic effect of butyrate (Fig. S1). These results are consistent with the finding that the individual propolis components did not affect significantly the levels of pAKT, pcJUN, or pSTAT3 in butyrate-treated CC cells (Fig. S1B).

## Discussion

The cancer preventive properties of individual diet-derived compounds, including butyrate and CAPE, have been examined; however, there are no studies that have evaluated the combined anti-neoplastic potential of the agents. Some, but not all, honeybee propolis preparations are the only source of CAPE in human diet; therefore, we investigated the combined effects of butyrate and CAPE-containing propolis on CC cells. We observed that similar to CAPE [3], CAPE-containing propolis augments butyrate-induced apoptosis of CC cells (Figs. 1B,C). One mechanism for this effect is the suppression of the AKT cell survival pathway, a major target of CAPE in butyrate-treated CC cells (3, and Fig. 1A). However, since propolis (providing 0.3 µg/ml CAPE) is more potent than CAPE (utilized at 1.2 µg/ml) in resensitizing butyrate-resistant HCT-R cells to butyrate-induced apoptosis (Fig. 1), we ascertained whether propolis affects cell survival pathways other than AKT signaling. Here we investigated the role of JNK signaling, a pathway that may contribute to compensatory cell survival in apoptotic cell populations. We established that exposure of CC cells to butyrate/propolis treatment activates the JNK enzymes by phosphorylation at amino acid residues T183 and Y185A (Fig. 2A), and that the inhibition of JNK signaling increases the apoptosis of drug-resistant HCT-R cells (Fig. 2C). Since the JNK pathway affects the phosphorylation status of cJUN and the signaling through JAK/STAT, we evaluated the role of these signaling molecules in the apoptosis of butyrate/propolis-treated CC cells.

While investigating the role of phosphorylated cJUN, we established that its dominant negative form, TAM67, renders HCT-116 cells more resistant to butyrate/propolis-induced apoptosis (Fig. 3E). A simple interpretation of these results is that endogenous cJUN has a moderate pro-apoptotic transcriptional activity, and this function is abrogated by TAM67. An alternative explanation, however, is based upon reports that cJUN has both pro-apoptotic and anti-apoptotic functions [36], and TAM67 may preserve only the activity of cJUN that supports resistance to apoptosis [37]–[39]. Our results corroborate this possibility: TAM67 not only suppresses the induction of AP1 transcriptional activity, but also decreases the hyperinduction of beta-catenin/TCF-dependent promoters by butyrate/propolis (Fig. 3D, and Fig. 3H,I). The effect of TAM67 on WNT transcriptional activity might be due to the ability of its leucine zipper domain to bind beta-catenin, as it has been reported for cJUN [26]. Binding between beta-catenin and TAM67 and/or decreased binding between beta-catenin and endogenous cJUN (Fig. 3G) may limit the transactivation of some promoters targeted by beta-catenin. Since hyperinduction of WNT/beta-catenin activity is required for high levels of butyrate-induced apoptosis [1]–[3], the suppression of WNT/catenin transcriptional activity by TAM67 (Fig. 3H,I) can counteract apoptosis. Complexes between TAM67 and STAT3 or TAM67 and Sp1 may also have an anti-apoptotic role [40], [41]. This explanation is congruent with the finding that silencing of c*JUN* does not affect apoptosis in butyrate/propolis CC cells (Fig. 3B): thus, c*JUN* silencing may suppress both, the pro-apoptotic and anti-apoptotic functions of the transcriptional factor; whereas, TAM67 may preserve only the anti-apoptotic transcriptional role of cJUN.

In HCT-116 cells, cJUN/beta-catenin/TCF4 complexes bind to approximately 400 genomic sites with proximal AP1 and TCF motifs [25], [42]; however, it is unknown how hyperactivation of cJUN and transcriptionally active beta-catenin by butyrate/propolis treatment of CC cells affects the targets of these complexes. Since butyrate/propolis exposure induces high levels of apoptosis, we posit that the increased levels of cJUN/beta-catenin/TCF4 complexes either induce pro-apoptotic genes that have not been reported previously, or hyperinduce the already known gene targets [42] and their overexpression leads to apoptosis. The significantly suppressed induction of a WNT/catenin promoter with TCF motifs (LEF-OT, Fig. 3H) might be due to the titration of beta-catenin/TCF4 complexes away from the targeted pro-apoptotic genes by TAM67, or to the direct inhibition of these genes by complexes between beta-catenin/TCF4 and TAM67. However, the inability of TAM67 to downregulate significantly the activity of a promoter with proximal AP1 and TCF motifs (TOPFlash, Fig. 3I), suggests that TAM67 may have a residual activity on some promoters. Future immunoprecipitation and chromatin immunoprecipitation-sequencing experiments can address this possibility.

Our results indicate that JAK/STAT signaling, another downstream target of the JNK pathway, supports survival of butyrate/propolis-treated CC cells. Our data are in agreement with observations that JAK/STAT3 signaling contributes to the viability of CC cells *in vitro,* a relevant finding since abnormal activation of this pathway is detected in primary colon cancers [43]. We observed that the addition of a JAK/STAT inhibitor (JAKi) to butyrate/CAPE- or butyrate/propolis-treated cells moderately augments apoptosis; however, exposure of CC cells to JAKi alone does not produce cell death (Figs. 4C and 4D). Therefore, JAK/STAT signaling acts as a true compensatory survival signaling pathway: it contributes to cell survival in apoptotic CC cell populations; however, its suppression in absence of an apoptotic inducer (e.g., butyrate) does not produce apoptosis in CC cells. Here we evaluated the phosphorylation status of only one major member of the JAK/STAT pathway, STAT3, and future studies should establish whether additional JAK/STAT signaling components are activated in butyrate/propolis CC cells.

Compared to HCT-116 cells, drug-resistant HCT-R cells express higher levels of pSTAT3, and propolis downregulates these levels (Fig. 2A). Whereas propolis downregulates both pAKT and pSTAT3 levels (Figs. 1 and 2), CAPE suppresses the levels of phosphorylated AKT, but not these of phosphorylated STAT3, in butyrate-treated HCT-R cells (Figs. 1A and 4A). Therefore, it is plausible that the inhibition of JAK/STAT signaling by propolis explains the more potent pro-apoptotic effect of propolis than that of CAPE on butyrate-treated HCT-R cells (Fig. 1B). This interpretation is consistent with the finding that adding a JAKSTAT inhibitor to butyrate/CAPE produces apoptotic levels comparable to these in butyrate/propolis-treated HCT-R cells (22.3±4.2% *vs*. 29±5.9%, Figs. 4C and 4D).

Our data suggest that New Zealand propolis (propolis with CYCLOPOWER ™, Manuka Health New Zealand Ltd) contains compound(s) that suppress JAK/STAT signaling (Figs. 2A and 4A); however, addition of a pharmacological inhibitor of the JAK/STAT pathway further augments apoptosis of butyrate/propolis-treated CC cells (Fig. 4C). Therefore, a dietary supplement containing fiber (a source of butyrate) and propolis could benefit from the addition of diet-derived JAK/STAT inhibitors, and several such compounds have been identified [44]–[46]. Our data also indicate that specific compounds in propolis contribute to its apoptosis-augmenting capability when combined with butyrate. Identification of these individual compounds will allow for control and standardization of propolis supplements. However, analyses on chrysin, pinocembrin, and galangin, all found in New Zealand propolis, did not support a role for these compounds in augmenting butyrate-induced apoptosis in CC cells (Fig. S1). Therefore, it is likely that different propolis compounds account for the effects of propolis and/or that the cumulative activity of several propolis compounds is required to observe the effect.

In all *in vitro* experiments we utilized a concentration of 5 mM butyrate, which is within the physiologically achievable colonic levels after consumption of dietary fiber [17]. The achievable levels of propolis in the colon are unknown. However, since humans can consume gram quantities of propolis, it is likely that a concentration of 100 ug/ml or greater is attainable in the colon. Thus, the effects we have observed *in vitro* are likely achievable *in vivo*. Furthermore, the activity of propolis may be influenced by other dietary components; therefore, it is essential to determine whether additional diet-derived bioactives augment the apoptotic effect of butyrate and propolis on CC cells by targeting all cell survival pathways. Such additional bioactives can be utilized to design a dietary supplement with high effect on decreasing the risk of CC.

**Figure 5:**
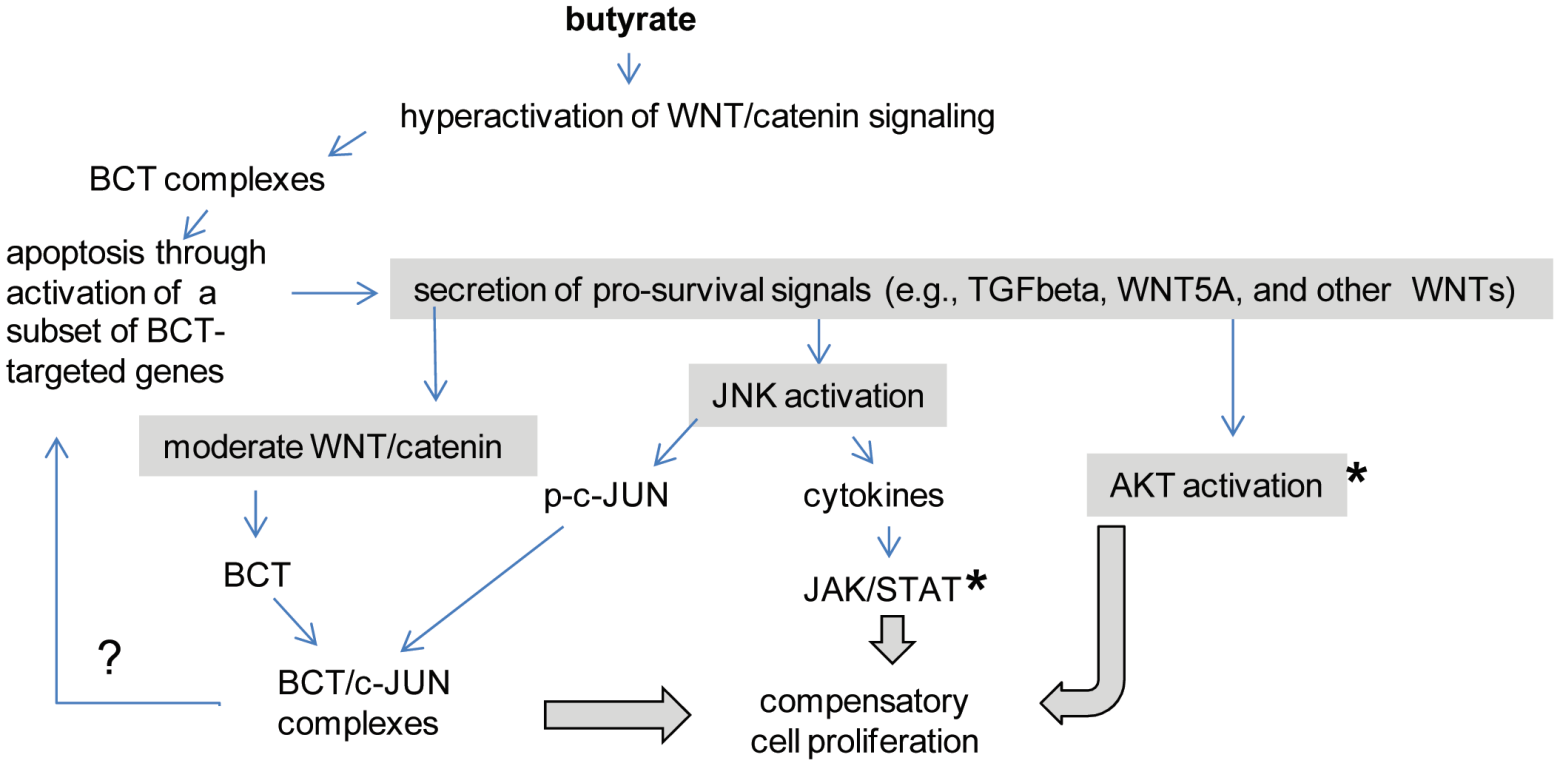
Compensatory survival signaling in apoptotic CC cell populations. Butyrate hyperinduces WNT/beta-catenin activity in CC cells with mutations in the pathway by increasing the formation of beta-catenin/TCF4 (BCT) complexes [1], [2]. High levels of BCT complexes induce directly pro-apoptotic genes such as BAX and BOK [47]. However, due to signaling heterogeneity, only a fraction of all cells hyperactivates the pathway and undergo apoptosis within 24 hours of exposure to butyrate [1]. Similar to cells undergoing compensatory proliferation [13], [15], apoptotic CC cell populations increase the expression of TGFbeta, WNT5A, and WNT11 [3], [16], and these molecules activate pro-survival pathways, such as JNK and AKT signaling [3], (Figs. 1,2). JNKs activate cJUN and JAK/STAT signaling [22], [23]. Inhibition of JNK augments apoptosis induced by butyrate/propolis treatment (Fig. 2C), as does the inhibition of its downstream target, the JAK/STAT pathway (Fig. 4). The role of pcJUN in the apoptotic response of CC cells to butyrate/propolis exposure is complex: at relatively lower levels, cJUN may contribute to proliferation; whereas, when hyperinduced together with beta-catenin/TCF complexes it may induce apoptosis. Asterisks mark the pathways inhibited by propolis.

The main aim of our future studies will be to determine whether dietary supplements of propolis and fermentable fiber lessen CC incidence in individuals with increased risk of this malignancy: patients with chronic inflammatory bowel syndrome, resected CC, positive colonoscopies, and obese individuals. A successful strategy in combining individual diet-derived bioactives in CC-preventive supplements could follow the approach of combining two modules: the first is an apoptosis-inducing module (e.g., fiber-derived butyrate, a powerful histone deacetylase inhibitor), and the second module consisting of inhibitors of cell survival pathways (e.g., AKT, JAK/STAT pathways). Our hypothesis is depicted in Fig. 5.

## Materials and Methods

### Cell Culture, Recombinant Plasmids, and Chemicals

The human CC cell line HCT-116 was obtained from the American Type Culture Collection (Rockville, MD). The cell line HCT-R was derived from HCT-116 by culturing the parental cells in increasing concentrations of butyrate as previously described [2]. CC cells were grown in alpha-MEM medium with 10% fetal bovine serum and antibiotics. Manuka Health New Zealand Ltd provided propolis with CYCLOPOWER ™, in which CAPE concentration is at 0.3 µg per 100 µg propolis. We utilized the WNT/beta-catenin luciferase reporters pTOPFLASH, with a minimal c-*FOS* promoter and with three copies of the optimal LEF/TCF-binding motif CCTTTGATC, and pFOPFLASH with a minimal c-*FOS* promoter and with three copies of the mutant LEF/TCF-binding motif CCTTTGGCC (a kind gift from Dr. H. Clevers, UMC Utrecht, Utrecht, Netherlands). These two luciferase reporter vectors also contain an AP1-binding motif (TGTCTC). The second pair of luciferase reporters for WNT/beta-catenin activity was LEF-OF and LEF-OT (Dr. B. Vogelstein, Johns Hopkins University, Baltimore, MD), and these two reporters combine the E1B TATA box promoter with the wild or mutant TCF-binding motifs described above. LEF-OT and LEF-OF do not have an AP-1 binding motif. We also used pGFP-TAM67 (kindly provided by Dr. R. Hennigan, Cincinnati Children’s Hospital Medical Center, Cincinnati,OH), and (AP-1)_4_ luciferase reporter (a gift from Dr. N. Colburn, Laboratory of Cancer Prevention Frederick National Lab, Frederick, MD). Sodium butyrate was obtained from Sigma, caffeic acid phenethyl ester and SP600125 from Santa Cruz Biotechnology. Stock solutions were prepared in dimethyl sulfoxide, except for butyrate, which was dissolved in water at 1 M concentration. Stable expression of the dominant negative cJUN protein TAM67-GFP in HCT-116 and HCT-R cells was accomplished by selection with G418.

### Transfections

Transfections were carried out with Lipofectamine 2000 (Life Technologies, Rockville, MD), or via nucleofection with Amaxa (Lonza). For the transcriptional reporter assays, we applied the reverse Lipofectamine transfection protocol. Complexes between DNA and Lipofectamine were pre-formed in a 96-well plate format, and cells were added at 50,000 per well. The vector pRSV-TK (Promega Corp., Madison, WI) was used for normalization of transfection efficiency in all luciferase reporter assays, which were performed using a Turner Luminometer and a Dual Luciferase kit (Promega, Madison, WI). Silencing of *c-JUN* was achieved with siRNA from Santa Cruz Biotechnology (sc-29223). One million HCT-R or HCT-116 cells were nucleofected with 180 pmol of *c-JUN* siRNA or control siRNA, and treated with 5 mM butyrate and 100 µg/ml propolis for 24 hours.

### Western Blot Analyses, Immunoprecipitations, and Antibodies

Western blot analyses were performed as reported previously [1]. The following antibodies were used: from Cell Signaling, anti-pJNK (#4668, recognizes the two JNK isoforms of 46 kD and 54 kD when phosphorylated at T183 and Y185), anti-pSTAT3 (#9145, recognizes STAT3 phosphorylated at Y705), and anti-pcJUN (#9261, recognizes the form phosphorylated at S63); from Santa Cruz Biotechnology, anti-Ser473-phosphorylated AKT (sc-7985), total c-JUN (sc-44 and sc-45) and anti-beta-catenin (sc-53483); from Sigma Aldrich, anti-ACTIN (A5441). Nuclear lysates were obtained with Nuclei EZ kit (Sigma Aldrich), and immunoprecipitations were carried out with 60 µg of nuclear protein and 2 µg of antibody to c-JUN (sc-45).

### Apoptotic Assays

Twenty-four hours prior to treatments CC cells were plated in 24-well plates at 100,000–120,000 per well. All cells (floating and attached) were harvested and stained for apoptotic and necrotic markers with PE Annexin V Apoptosis Detection Kit I (BD Biosciences, #559763). Flow cytometry analyses were carried out with FACS Aria II and DiVa software. Percent apoptosis is the number or apoptotic cells divided by the number of all analyzed cells, and multiplied by 100. Fold induction of apoptosis is the ratio of percent apoptotic cells in butyrate/propolis-treated samples to percent apoptotic cells in mock-treated samples.

### Statistics

All data were presented as mean ± standard deviation from at least three sets of independent experiments. Student T-test analysis was used to determine the significance of statistical differences. Differences were considered significant at P<0.05.

## Supporting Information

Figure S1 **Analyses on the role of individual propolis compounds in butyrate-treated CC cells.** (A). A representative western blot analysis of HCT-R cells exposed to mock (M), 5 mM butyrate (B), butyrate and 3 µM chrysin (B/C), butyrate and 7 µM pinocembrin (B/P), or butyrate and 2 µM galangin (B/G) for 19 h. (B) A representative apoptotic assay with three samples per treatment. Cells were exposed to the treatments described in (A) for 48 h, and analyzed for apoptosis via a flow cytometry assay.
(TIF)
