# Supplementary figures and images for: Propolis Augments Apoptosis Induced by Butyrate via Targeting Cell Survival Pathways
Source: PLoS One. 2013 Sep 4;8(9):e73151. doi: 10.1371/journal.pone.0073151 (PMC3762847; doi:10.1371/journal.pone.0073151)

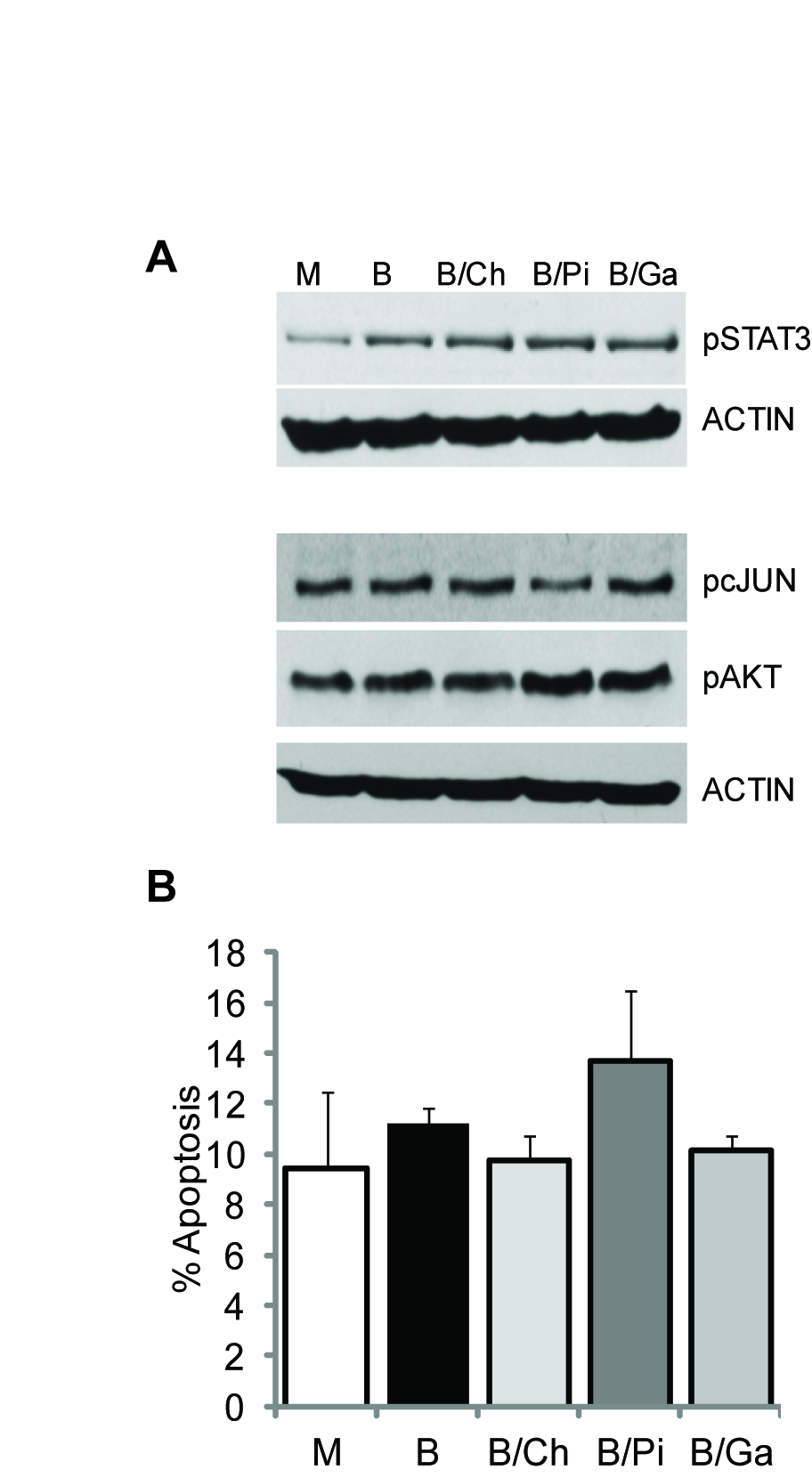

Supplement: Figure S1 — Analyses on the role of individual propolis compounds in butyrate-treated CC cells. (A). A representative western blot analysis of HCT-R cells exposed to mock (M), 5 mM butyrate (B), butyrate and 3 µM chrysin (B/C), butyrate and 7 µM pinocembrin (B/P), or butyrate and 2 µM galangin (B/G) for 19 h. (B) A representative apoptotic assay with three samples per treatment. Cells were exposed to the treatments described in (A) for 48 h, and analyzed for apoptosis via a flow cytometry assay. (TIF) [file pone.0073151.s001.tif]
